# To which extent do breast cancer survivors feel well informed about disease and treatment 5 years after diagnosis?

**DOI:** 10.1007/s10549-020-05974-y

**Published:** 2020-10-26

**Authors:** S. L. Herbert, A. Wöckel, R. Kreienberg, T. Kühn, F. Flock, R. Felberbaum, W. Janni, C. Curtaz, M. Kiesel, T. Stüber, J. Diessner, J. Salmen, L. Schwentner, V. Fink, I. Bekes, E. Leinert, K. Lato, A. Polasik, F. Schochter, S. Singer, BRENDA study group

**Affiliations:** 1 Department of Obstetrics and Gynaecology, University Medical Centre Würzburg, Würzburg, Germany; 2 Department of Gynaecology and Obstetrics, University of Ulm, Ulm, Germany; 3 Department of Gynaecology and Obstetrics, Hospital Esslingen, Esslingen, Germany; 4 Department of Gynaecology and Obstetrics, Hospital Memmingen, Memmingen, Germany; 5 Department of Gynaecology and Obstetrics, Hospital Kempten, Kempten, Germany; 6 Institute of Medical Biostatistics, Epidemiology and Informatics, University Medical Centre Mainz, Mainz, Germany

**Keywords:** Breast cancer, Survivors, Unmet needs, Health care providers

## Abstract

**Objective:**

In this study, we investigated to which extent patients feel well informed about their disease and treatment, which areas they wish more or less information and which variables are associated with a need for information about the disease, medical tests and treatment.

**Methods:**

In a German multi-centre prospective study, we enrolled 759 female breast cancer patients at the time of cancer diagnosis (baseline). Data on information were captured at 5 years after diagnosis with the European Organisation for Research and Treatment of Cancer (EORTC) Information Module (EORTC QLQ-INFO24). Good information predictors were analysed using linear regression models.

**Results:**

There were 456 patients who participated at the 5-year follow-up. They reported to feel well informed about medical tests (mean score 78.5) and the disease itself (69.3) but relatively poorly about other services (44.3) and about different places of care (31.3). The survivors expressed a need for more information concerning: side effects and long-term consequences of therapy, more information in general, information about aftercare, prognosis, complementary medicine, disease and therapy. Patients with higher incomes were better informed about medical tests (β 0.26, *p* 0.04) and worse informed with increasing levels of fear of treatment (β − 0.11, *p* 0.02). Information about treatment was reported to be worse by survivors > 70 years old (β -0.34, *p* 0.03) and by immigrants (β -0.11, *p* 0.02). Survivors who had received additional written information felt better informed about disease, medical tests, treatment and other services (β 0.19/0.19/0.20/0.25; each *p* < 0.01).

**Conclusion:**

Health care providers have to reconsider how and what kind of information they provide. Providing written information, in addition to oral information, may improve meeting those information needs.

## Introduction

Brest cancer is the most common cancer among women worldwide. Aside from medical treatment of cancer, the patients’ mental health is of great importance. Since these women are confronted with many challenges in dealing with physical symptoms, side effects, anxiety and fear of recurrence, all of which leading to a reduced quality of life, the patients´ well-being has to be focused [1]. These conditions evoke supportive needs. The number of breast cancer survivors is increasing, but unfortunately, there seems to be a lack of good long-term supportive care. Important issues are inadequately addressed [2]. Hence, in this study, we investigated unmet needs of breast cancer long-term survivors.

One of the most important supportive factors is information [3]. Studies have shown greater need for information especially among patients with breast cancer [4]. Need for information is the most frequent unmet need in all phases of the disease [5]. There is a high demand for structured and detailed information on the one hand and a different perception of the healthcare providers regarding the patients´ needs on the other hand [6]. Obviously, these circumstances lead to a discrepancy between patients and healthcare providers concerning the patients need for information and what they are provided with. A significant burden for receiving information is created [6], since patients have to look for other sources of information by themselves. Not every patient needs the same kind of information. It can be varied by different factors such as gender, age, educational level and coping style [7, 8]. Information can counteract the patients’ helplessness, which gives them a feeling of control [3]. Meeting their information needs can support self-management strategies of patients [9]. This enables choosing support offers. Increased well-being can lead to a better quality of life [10]. In contrast, confusing information can reduce quality of life for a long period of time [11]. A lack of information can affect mental and physical well-being. Depressive symptoms, anxiety and lower functioning levels can be the consequence. The illness perception is influenced by these conditions. It is known that patients form their beliefs of illness based on information. These beliefs are part of the self-regulation [12]. In this way, healthcare providers can either support or obstruct the mechanism of self-regulation.

There are many other benefits of providing information such as informed decision making, a lower stress level, better treatment adherence, improved satisfaction and a sense of control for the patients [13, 14]. Since the need of information is of major importance to the well-being and compliance for therapies despite the side effects, we need more data for providing a well-adapted level of information for the patients.

Therefore, this study has three purposes to find out (I) whether the patients consider themselves well informed; (II) what areas of care they want to know more/ less about; (III) what group of patients feel well informed about their illness and treatment. We hypothesised that the following variables are associated with a sense of being well informed: age and fear of treatment.

## Methods

### Study design

We conducted a prospective multi-centre study (BRENDA II). Breast cancer patients were enrolled at the time of their cancer diagnosis at four hospitals. All participating clinics (*n* = 4) were certified breast cancer centres. Patients were eligible for this study if they had been diagnosed with primary breast cancer, were female, able to complete questionnaires and had provided written informed consent. Exclusion criteria were metastatic disease, recurrent disease, bilateral breast cancer, primary occult disease and phylloides tumour. Five years later (5Y), patients were contacted again, and we ascertained how well informed they felt about the disease, its treatment and aftercare. The Ethics Committee of Ulm University approved the study.

### Instruments

#### Information

Data were captured with the EORTC Information Module (EORTC QLQ-INFO25) [15]. The EORTC QLQ-INFO25 questionnaire asks how much information cancer patients received about illness and treatment, if information was distributed via CD/ video or written, if patients requested further information and if they found information helpful as a whole. In total, the instrument contains 25 items with a Likert response scale. The first 17 items are aggregated into four multi-item scales (information about disease, medical tests, treatment, other services) according to the scoring manual of the EORTC [16]. A score is calculated if at least half of all items per scale are completed. The raw values are transformed on a scale from 0 to 100. The higher the score is, the better informed the patients feel. The other items form the so-called single-item scales. They are composed of one item and are transformed from 0 to 100 as well.

#### Demographic data

Education was categorised with the highest school leaving qualification. The income was calculated using an equalised income (OECD scale). Patients with a migration background were defined as not born in Germany, no German citizenship or no German nationality.

#### Clinical data

The stage of disease was obtained from the medical charts. It was classified in locally advanced disease versus not locally advanced (≥ T2, N1).

#### Mental health

Mental health was assessed with the long form of the Patient Health Questionnaire (PHQ) [17].

#### Fear of treatment

Patients were asked at baseline how afraid they were concerning their surgery, lymph node dissection, radiotherapy, chemotherapy and endocrine treatment. These items were summarised into a total score for “fear of treatment” with 1 indicating no fear at all; and 4 indicating very strong fear of treatment. Cronbach’s Alpha was 0.81.

### Statistical analysis

To answer the first two research questions, we used descriptive analyses (absolute and relative frequencies, means).

We conducted a multivariate linear regression analysis for answering the third question. As it is known that the relationship between fear and several outcomes is often u shaped (i.e., very low levels of fear and very high levels of fear have similar effects), we checked this assumption against a LOWESS plot. However, the plot indicated that the relationship was fairly linear.

The following predictors were entered into the regression model: age (< 40, 41–50, 51–60, 61–70, > 70 years), education (< vs. ≥ 10 years), income (equivalent income according to the OECD), migration status (yes/no), locally advanced disease (≥ T2, N1), comorbid psychiatric disease (yes/no), fear of treatment, mastectomy vs. breast conserving surgery, received radiotherapy (yes/no), received chemotherapy (yes/no) and received endocrine therapy (yes/no).

## Results

### Sample—description

In this study, we enrolled 759 patients. At the five-year follow-up, 456 (60%) patients participated again. 60 patients were deceased; 101 declined participation; 1 had moved to an unknown place and could not be traced and 141 could not be contacted because of logistic reasons.

Patients who rejected participation at 5Y but took part in the study before were on average 7.8 years older than the patients who participated at 5Y.

The average age of the participants was 64 years at the time of 5Y (SD 11 years). Nearly half of the participants had less than 10 years of education (43%). 41% had more than 1500 € income per person per month. 14% were immigrants. 20% of the participants had psychiatric comorbidities. A locally advanced disease was found in 54% of the cases. 86% of the participants had a breast conserving surgery, and 14% had a mastectomy. 91% underwent radiotherapy, 46% chemotherapy and 82% endocrine therapy (Table 1). Anxiety of treatment was on average 2.8 at t1 (SD 0.7).

**Table 1:** Sample characteristics – distribution of characteristics of participants in percent (%)

		N	%
Age at 5Y after diagnosis (in years)	< 40	8	2%
	40–49	50	11%
	50–59	131	29%
	60–69	123	27%
	70–79	115	25%
	80 +	19	4%
	Unknown	10	2%
Education in years	< 10	195	43%
	> = 10	256	56%
	Unknown	5	1%
Income in € per person per month	< 500	18	4%
	500 to 999	87	19%
	1000 to 1499	102	22%
	> 1500	186	41%
	Unknown	63	14%
Immigrant	No	387	85%
	Yes	64	14%
	Unknown	5	1%
Psychiatric comorbidity	No	361	79%
	Yes	89	20%
	Unknown	6	1%
Locally advanced disease	No	209	46%
	Yes	247	54%
Surgical treatment	Breast conserving	392	86%
	Mastectomy	64	14%
Radiotherapy	No	40	9%
	Yes	416	91%
Chemotherapy	No	247	54%
	Yes	209	46%
Endocrine therapy	No	84	18%
	Yes	372	82%

### Being informed—description

Most patients reported being very much or quite informed about medical tests (mean score 78.5) and the disease (mean 69.3).

Compared to these two domains, patients felt less informed about treatment (57.1), about things they can do to help themselves (59.6), about other services (44.3) and different places of care (31.3).

There was quite a high level of satisfaction with information (70.1) and overall information have been helpful (74.0).

A large part of the patients (69%) had received written information, and 9% had received information on CD/video.

About a quarter of the participants (*n* = 101, 24%) said they would like to receive more information, and 2% wished to receive less information. All patients had the chance to write what they wanted to know more or less about. 93 of them gave an answer. 25.5% of them wished to get more information about side effects and long-term consequences of therapy; 15.1% asked for more information in general and 12.9% needed more information about aftercare. About 5% wanted to receive more information about prognosis, complementary medicine, disease and therapy.

### Predictors of being informed

Patients felt less informed concerning treatment with increasing age (see Table 2). Especially the patients in the two highest age groups (70 years and older) reported lower information levels (β −0.34 *p* = 0.03 and β −0.29, *p* < 0.01)). The highest age group (80 + years) also felt less informed about other services (β −0.14. *p* = 0.08).

**Table 2:** Predictors of being informed—standardised β coefficients from multivariate linear regression analyses

	Information provided	Disease		Medical tests		Treatment			Other services		
		Stand. β	*p*-value	Stand. β	*p*-value	Stand. β		*p*-value	Stand. β		*p*-value
Age at 5Y in years	< 40	Reference									
	40–49	0.20	*0.11*	− 0.01	*0.91*	− 0.08		0.49	0.05		0.69
	50–59	0.24	*0.17*	− 0.03	*0.88*	− 0.20		0.24	0.04		0.82
	60–69	0.23	*0.17*	0.00	*1.00*	− 0.19		0.25	− 0.04		0.83
	70–79	0.07	*0.67*	− 0.17	*0.29*	− 0.34		0.03	− 0.22		0.16
	80 +	− 0.04	*0.64*	− 0.08	*0.35*	− 0.29		0.00	− 0.14		0.08
Education in years	< 10	Reference									
	> = 10	0.05	*0.39*	0.07	*0.21*	0.04		0.41	− 0.01		0.79
	Unknown	0.00	*0.94*	0.01	*0.89*	0.00		1.00	− 0.01		0.89
Income in € per person per month	< 500	Reference									
	500 to 999	− 0.02	*0.88*	0.08	*0.47*	− 0.15		0.19	− 0.10		0.34
	1000 to 1499	0.01	*0.92*	0.16	*0.16*	− 0.10		0.38	− 0.07		0.55
	> 1500	0.04	*0.75*	0.26	*0.04*	− 0.14		0.31	− 0.13		0.30
	Unknown	− 0.03	*0.78*	0.07	*0.42*	− 0.12		0.22	− 0.09		0.32
Migration status	Immigrant	− 0.06	*0.27*	− 0.06	*0.26*	− 0.11		0.02	− 0.11		0.02
Psychiatric comorbidity	No	Reference									
	Yes	− 0.08	*0.13*	− 0.05	*0.32*	− 0.05		0.30	0.05		0.37
	Unknown	0.08	*0.11*	0.06	*0.22*	0.08		0.09	− 0.02		0.63
Anxiety	(in score points)	− 0.08	*0.13*	− 0.11	*0.02*	− 0.04		0.44	− 0.06		0.23
Disease status	Locally advanced	0.03	*0.61*	0.00	*0.96*	0.01		0.92	− 0.01		0.82
Surgical treatment	Breast conserving	Reference									
	Mastectomy	− 0.08	*0.19*	0.02	*0.69*	− 0.03		0.58	− 0.03		0.59
Adjuvant treatment	Radiotherapy	− 0.07	*0.25*	− 0.04	*0.44*	− 0.02		0.69	− 0.01		0.86
	Chemotherapy	− 0.02	*0.77*	− 0.01	*0.87*	0.05		0.39	0.01		0.89
	Endocrine therapy	− 0.03	*0.61*	− 0.03	*0.57*	− 0.02		0.75	− 0.03		0.58
R^2^		0.08	*0.02*	0.09	< *0.01*	0.12		< *0.01*	0.10		< *0.01*

Italics indicate significance of *p*-value: 0.05

Concerning information about disease, no evidence for differences between age groups was found.

A higher income was associated with a higher level of feeling informed about medical tests (β 0.26, *p* = 0.04). This difference was not observed in information about the disease, treatment and other services.

There was no evidence for an effect of education on the information level. Migrants felt significantly less well informed about the treatment (β − 0.11, *p* = 0.02) and other services (β −0.11, *p* = 0.02).

We did not find evidence for an effect of comorbid psychiatric diseases on the level of information. However, higher levels of fear of treatment are related to feeling less well informed about medical tests (β −0.11, *p* 0.02). The higher the fear of treatment patients had at the time of diagnosis, the lesser the information about treatment they had (or remembered to have received).

There was no evidence for an association between disease status, type of surgical treatments, the various adjuvant therapy strategies and the level of information (Table 2).

### Differences of being informed concerning providing information

Exploratorily, we investigated whether the type of information received (only oral or written or on CD/ video) is associated with the level of information. The majority of patients (*n* = 264) had received written information; 37 had received written information plus information on CD or video; 3 had received information on CD but not in writing and 130 had received neither written nor CD-based information.

There was evidence that those patients who were provided with written information (in combination with CD or only printed material) were feeling better informed about disease, medical tests, treatment and other services (β 0.16 to 0.25, *p* < 0.01) compared to the ones who did not receive anything in writing.

## Discussion

Since the need for information is of great importance for the patient’s well-being, it is indispensable to evaluate whether information provision meets the patient’s needs.

5 years after diagnosis, satisfaction with information is quite good. But there are still unmet needs. This is already known for other cancer survivors [18, 19]. Our data show unmet needs of information. Especially there are unmet needs about different places of care, other services and also things you can do to help yourself and treatment compared to information about the disease and medical tests. Since cancer survivors can be affected by physical, psychological and social problems [20], it is not surprising that unmet needs exist. These problems are of an individual character which underlines that an individual approach is necessary [21].

The first step of improving aftercare concerning unmet information needs is to realise the importance of satisfaction with information. It is already known that dissatisfaction with information can cause anxious and depressive symptoms [22], which makes improving the provision of information important. But there is more about anxiety than negative effects. A certain level of anxiety could be helpful for gaining and keeping information. This might indicate that there is a useful level of tension. Krohne already described this principle in his model of coping with fear [23]. One of the central dimensions that describes individual differences for coping with stress and fear is vigilance. The state of vigilance results in stronger inclusion and comprehensive processing of threatening information. Besides vigilance, the other basic construct of behavioural patterns is cognitive avoidance. Patients who are coping with cognitive avoidance avert their attention from threatening stimuli. Processing that kind of information is inhibiting. The explanation for reacting with vigilance or cognitive avoidance is the basis of developing fear. There is rising physical arousal in experiencing increasing insecurity [24]. Krohne’s model assumes that physical arousal leads to cognitive avoidance and increased insecurity, to vigilance which correlates with different personalities. The level of avoidance or vigilance depends on the personal tolerance of arousal or insecurity. This tells us that no matter how much information is provided, some patients cannot be well informed until health care providers help them cope with fear. Nevertheless, the correlation between fear and vigilance for information is not endless. The literature reports that there is a reverse correlation in patients with a higher level of fear. If fear rises beyond a certain level, the patients’ feeling of being informed is decreasing. At this point, a vicious cycle can emerge.

The next step is identifying problems concerning providing information for breast cancer survivors. In our study, there was evidence for a difference in the need for information about the treatment concerning the age. Patients of advanced age (70 years +) feel less informed about their treatment. There was no evidence that age is related to information about the disease, tests or other services. The reason for this phenomenon might be the mental fitness of the oldest group, it could be related to real cognitive problems, or the doctors simply assume they cannot understand everything. This, in turn, is reflected in our data. Another explanation might be specific needs of younger patients. Younger breast cancer patients are generally of reproductive age, which means they have specific issues [25]. Since adjuvant therapy causes premature menopause by chemotherapy and hormonal therapy, fertility is one of these issues [26]. Besides fertility, premature menopause and psychological problems are important topics as follow-up to these patients [27]. Breast cancer occurs more frequently in patients of higher age. Consequently, many of the younger patients feel that they are too young to develop cancer [28]. This might lead to the wish for receiving more information compared to older patients. This also matches with our data which shows a lack of information concerning treatment and the wish of being more informed about side effects and long-term consequences of therapy. A study from Turkey came to the same conclusion [29]. Most of the patients require endocrine therapy, affecting them for many years in different forms.

We would like to mention a few limitations of our study that should be kept in mind in order to evaluate its results. First, this was a prospective study with a 5-year follow-up. Like in most studies with such a long duration, participants drop out from the study for various reasons (death, decreasing motivation, decreasing health etc.) [30–32]. Those who drop out usually differ from those who continue to participate, and this was true for our study too. We identified that attrition was related to higher age. Hence, if age is related to information needs (and indeed this is what we found in those who participated, at least regarding information about treatment) than the mean information needs in the clinical population most likely differ somewhat from the ones we found in our study. Hence, the descriptive results must be interpreted with caution. Another limitation is we collected data on information needs only at follow-up and not at baseline. Hence, we cannot say anything about the course of information needs. The fact that we only ask for information needs about disease, treatment and medical tests in general is also a limiting factor. Participants had the possibility to write down about what they would like to receive more information in a free text area. But there were no systematic and detailed questions about information needs. Hence, we cannot say exactly which kind of information the patients would like to receive additionally. More detailed data about information needs would have been interesting and helpful to improve information provision more precisely.

At the end, health care providers have to think of how to improve information provision. Individualisation is a central aspect. We have to ask about the patients´ needs. Knowledge of these needs enables medical staff to provide the required information. Our data may also suggest providing additional written information can be helpful in order to reduce information overload [33]. With a rising level of need for information on the one hand and medical resource scarcity concerning time on the other hand, it ought to be discussed how sufficient additional information can be provided. Medical staff such as breast care nurses can be considered.

## Conclusion

Despite a good level of information in general, there is a lack of information for certain medical and especially non-medical questions. Since there is evidence that unmet needs can affect cancer survivors´ everyday life in a negative way, we should improve how information is provided. One option is using different channels such as written information. But it is not enough just to focus on how we can provide it. Patients of different ages have different needs regarding information. Health care providers have to differentiate and assess the needs of patients and be aware of the fact that non-medical issues are of importance for the patients.

A cancer diagnosis is accompanied with anxiety which is normal. But a high level of anxiety inhibits the ability of information processing. Reducing anxiety via early psycho-oncological care can be the key to meeting the informational needs of patients with a high level of anxiety.
